# A Preliminary List of Horizontally Transferred Genes in Prokaryotes Determined by Tree Reconstruction and Reconciliation

**DOI:** 10.3389/fgene.2017.00112

**Published:** 2017-08-28

**Authors:** Hyeonsoo Jeong, Arshan Nasir

**Affiliations:** ^1^Department of Animal Sciences, University of Illinois at Urbana-Champaign Urbana, IL, United States; ^2^Department of Biosciences, COMSATS Institute of Information Technology Islamabad, Pakistan; ^3^Evolutionary Bioinformatics Laboratory, Department of Crop Sciences, University of Illinois at Urbana-Champaign Urbana, IL, United States

**Keywords:** phylogenetics, horizontal gene transfer, bacterial evolution, tree reconstruction, tree reconciliation

## Abstract

Genome-wide global detection of genes involved in horizontal gene transfer (HGT) remains an active area of research in medical microbiology and evolutionary genomics. Utilizing the explicit evolutionary method of comparing topologies of a total of 154,805 orthologous gene trees against corresponding 16S rRNA “reference” trees, we previously detected a total of 660,894 candidate HGT events in 2,472 completely-sequenced prokaryotic genomes. Here, we report an HGT-index for each individual gene-reference tree pair reconciliation, representing the total number of detected HGT events on the gene tree divided by the total number of genomes (taxa) member of that tree. HGT-index is thus a simple measure indicating the sensitivity of prokaryotic genes to participate (or not participate) in HGT. Our preliminary list provides HGT-indices for a total of 69,365 genes (detected in >10 and <50% available prokaryotic genomes) that are involved in a wide range of biological processes such as metabolism, information, and bacterial response to environment. Identification of horizontally-derived genes is important to combat antibiotic resistance and is a step forward toward reconstructions of improved phylogenies describing the history of life. Our effort is thus expected to benefit ongoing research in the fields of clinical microbiology and evolutionary biology.

## Introduction

Prokaryotic organisms often exchange genetic material directly with other organisms not necessarily related by vertical descent (Soucy et al., 2015). This phenomenon, commonly referred to as horizontal gene transfer (HGT), plays important roles in bacterial evolution by mediating, for example, transfer of antibiotic resistance and virulence, and genes involved in metabolism and stress response among related and non-related prokaryotic species (Ochman et al., 2000; Soucy et al., 2015). HGT therefore remains a major obstacle in slowing down or stopping the emergence of multidrug resistant bacterial pathogens and its global impact on prokaryote evolution and human health and economy continues to be investigated (von Wintersdorff et al., 2016).

Accurate detection of HGT through bioinformatics approaches however remains a technical and conceptual challenge (Ravenhall et al., 2015). To overcome the challenge of genome-wide global detection of HGT events in prokaryotes, we recently developed a sequence analysis pipeline and database to detect and store pre-calculated HGT events for a total of 2,472 completely sequenced prokaryotic genomes (156 Archaea and 2,316 Bacteria; Jeong et al., 2016). Unlike the existing HGT detection methods that rely on evaluating either genomic content bias across species (e.g., GC or codon usage; Becq et al., 2010) or methods that detect phylogenetically atypical signatures (Podell et al., 2008), we performed an explicit evolutionary study by reconstructing “approximate” maximum likelihood (ML) (Price et al., 2010) gene trees for a total of 154,805 putatively orthologous gene sets (putative since we test their HGT sensitivity in downstream analysis) detected in 2,472 genomes and reconciling the gene trees against corresponding reference species trees built from 16S ribosomal RNA (rRNA) gene alignments. Incongruence between gene and species tree pairs was evaluated for three likely scenarios, (i) gene duplication, (ii) HGT, and (iii) gene loss (known as the DTL reconciliation, Bansal et al., 2012), out of which the most parsimonious reconciliations supported by HGT were kept in the online *HGTree* database available from http://hgtree.snu.ac.kr.

*HGTree* is a rich resource of pre-calculated HGT events in prokaryotic genomes and offers a fast and reliably utility for users to explore HGT quality and quantity in their own genomic datasets. While, we already studied the fraction of genes in prokaryotic genomes that participate in HGT previously (see Figure 3 in Jeong et al., 2016), we did not study the HGT sensitivity of each individual prokaryotic gene. In this study, we therefore retrieved the pre-calculated HGT events along with corresponding gene and reference (i.e., 16S rRNA) tree pairs from *HGTree* and studied the sensitivity of each gene to participate or not participate in HGT. HGT sensitivity was given by an HGT-index simply indicating the total number of detected HGT events on a gene tree when reconciled with the corresponding 16S rRNA tree and divided by the size of the gene set in terms of number of member genomes. Following this simple statistic, and keeping in mind the associated limitations (see Section Discussion), we produced lists of frequently and non-frequently transferred genes in prokaryotic genomes (Datasets S1, S2). Further, we also mapped the identified genes to protein family (Punta et al., 2012) and functional annotations (Gene Ontology Consortium, 2015) to yield deeper insights into the nature of horizontal genetic exchange being mediated by microorganisms.

## Results

We retrieved a total of 660,894 candidate HGT events detected on a total of 154,805 orthologous gene-reference tree pair reconciliations from the *HGTree* database (Jeong et al., 2016). Based on the total number of detected HGT events on each gene-reference pair reconciliation and the size of the gene set (i.e., total number of genomes or taxa), we calculated an HGT-index to indicate the sensitivity of each gene to participate or not participate in HGT (Datasets S1, S2). Mathematically, the HGT-index ranges from 0 (i.e., no HGT) to higher values, although <1 in all observed cases (Datasets S1, S2). In other words, no gene was “universally” exchanged horizontally among all genomes.

Out of the total 154,805 gene sets, we excluded 37,460 (24%) gene sets where either all pair-wise neighbor-joining (NJ) distances were close to zero, genomes include only one species, or harbored <10 genomes, from the downstream analysis. Of the remaining, 69,365/117,345 (59%) gene sets were sufficiently large (i.e., >10 genomes and <50% of available genomes, see Section Discussion) and yielded detectable gene-species tree conflict indicative of horizontal evolution (hereafter “HGT-genes,” Dataset S1). Notably, a total of 47,980 genes (41%) reported no detectable conflict during gene and species trees reconciliations and thus tentatively could be termed “HGT-free” genes (Dataset S2). Unsurprisingly, most of the HGT-free gene sets included very few genomes (<82 genomes) belonging to only a specific division of bacteria indicating they either evolved recently via vertical inheritance or were more likely to be exchanged among closely related members of the same species that do not result in topological tree conflict and were thus missed by our phylogeny-based method of HGT detection (Jeong et al., 2016).

Among the HGT-genes, the top-twenty genes with the highest HGT-indices included interesting proteins (Table 1). For example, the 30S ribosomal subunit protein S21 (Pfam Id: PF01165) had an HGT-index of 0.80 (153 HGT events in 191 taxa) indicating it was widely transferred. Interestingly, the 30S smaller ribosomal subunit of prokaryotic ribosomes houses the 16S rRNA and 22 ribonucleoproteins. The smaller subunit is important for maintaining the accuracy and continuity of protein translation and is an active inhibition site for routinely used antibiotics such as tetracyclines and aminoglycosides (Mehta and Champney, 2002). The antibiotics typically bind to the 30S subunit and block protein synthesis ultimately leading to bacterial death. Similarly, another smaller subunit protein (S18) (PF01084) had an HGT-index of above 0.6 for a total of 193 HGT events in 319 taxa (Dataset S1). Within the top-twenty list was also the protein family aminoglycoside/hydroxyurea antibiotic resistance kinase (PF04665) that blocks aminoglycoside antibiotics (e.g., streptomycin) by phosphorylation. Streptomycin also acts by blocking the 30S ribosomal subunit and is in fact one of the most essential medicines recognized by the World Health Organization (WHO Model List of Essential Medicines 18th list, 2013). In addition, we also identified phage integrases (PF00589) and transposases (PF02371 PF01548) protein families that had very high HGT-indices (>70%) highlighting the roles of mobile elements in spreading genes among prokaryotic species. However, in these two cases, the orthologous gene sets were very small in size and contained only 28 taxa each from Proteobacteria (20 detected HGTs) (Dataset S1).

**Table 1:** Pfam Ids and descriptions for top-20 HGT-genes with the highest calculated HGT-index.

**PFAM Ids**	**PFAM Descriptions**	**# Genomes**	**# HGTs**	**HGT-index**	**Phyla**
PF01165	Ribosomal protein S21	191	153	0.801	Proteobacteria
PF00589, PF13356	Phage integrase family, Domain of unknown function (DUF4102)	28	20	0.714	Proteobacteria
PF02371, PF01548	Transposase IS116/IS110/IS902 family, Transposase	28	20	0.714	Proteobacteria
PF00137	ATP synthase subunit C	187	127	0.679	Proteobacteria
PF00126, PF03466	Bacterial regulatory helix-turn-helix protein, lysR family, LysR substrate binding domain	63	42	0.667	Proteobacteria
PF04655	Aminoglycoside/hydroxyurea antibiotic resistance kinase	32	21	0.656	Actinobacteria, Proteobacteria
PF04432, PF12838	Coenzyme F420 hydrogenase/dehydrogenase, beta subunit C terminus, 4Fe-4S dicluster domain	29	19	0.655	Bacteroidetes, Euryarchaeota, Firmicutes, Proteobacteria, Spirochaetes
PF00584	SecE/Sec61-gamma subunits of protein translocation complex	17	11	0.647	Proteobacteria
PF04397, PF00072	LytTr DNA-binding domain, Response regulator receiver domain	14	9	0.643	Bacteroidetes
PF09035	Excisionase from transposon Tn916	28	18	0.643	Firmicutes
PF13175	AAA ATPase domain	50	32	0.640	Actinobacteria, Bacteroidetes, Euryarchaeota, Firmicutes, Fusobacteria, Planctomycetes, Proteobacteria
PF01322	Cytochrome C'	11	7	0.636	Proteobacteria
PF12645	Helix-turn-helix domain	22	14	0.636	Firmicutes
PF09278, PF00376	MerR, DNA binding, MerR family regulatory protein	52	33	0.635	Nitrospirae, Proteobacteria
PF11990	Protein of unknown function (DUF3487)	38	24	0.632	Proteobacteria
PF01872	RibD C-terminal domain	43	27	0.628	Actinobacteria, Chloroflexi, Proteobacteria
PF11190	Protein of unknown function (DUF2976)	40	25	0.625	Proteobacteria
PF03795	YCII-related domain	64	40	0.625	Actinobacteria
PF07508, PF13408, PF00239	Recombinase, Recombinase zinc beta ribbon domain, Resolvase, N terminal domain	80	50	0.625	Actinobacteria, Chloroflexi, Euryarchaeota, Firmicutes, Spirochaetes, Tenericutes, Thermotogae
PF10502	Signal peptidase, peptidase S26	34	21	0.618	Proteobacteria

*Multiple protein family ids and descriptions are separated by comma. The top-20 list does not include genes for which no Pfam assignment was detected. See Dataset S1 for full list*.

Because the likelihood of HGT events increases for larger gene trees, next we studied the HGT-indices of genes harboring the maximum number of taxa (note that we excluded gene sets harboring >50% of genomes as their processing was computationally intensive and HGT event assignments were conceptually challenging; Table 2). Remarkably, the second-highest scoring hit belonged to the S13/S18 proteins of the 30S ribosomal subunit (PF00416) for which a total of 245 HGT events were detected in 915 taxa (HGT-index = 0.27) belonging to 11 major bacterial divisions indicating widespread transfer. The top-20 list also included fructose-1,6-bisphosphatase, an enzyme involved in cold adaptation and hibernation in mammals (Storey, 1997), other metabolic enzymes, and the S18 and (L13 at #23) proteins structural constituents of ribosomes. Interestingly, the majority of these transfers included many bacterial phyla (Table 2). Performing a keyword count on the annotated biological process gene ontology (GO) terms (Ashburner et al., 2000) revealed that most of the HGT-genes were involved in *metabolic* (*n* = 3,582 words), *transport* (3,565), *transcription* (2,766), *transduction* (1,791), and *translation* (875), along with *antibiotic* and *toxin* (27 each), *stress* (285), *homeostasis* (221), *rRNA* (211), and *transposition* (174) processes indicating the wide range of functions mediated by HGT in prokaryotes.

**Table 2:** Pfam Ids and descriptions are given for top-twenty HGT-genes with the highest number of genomes.

**PFAM Ids**	**PFAM Descriptions**	**# Genomes**	**# HGTs**	**HGT-index**	**Phyla**
PF01136	Peptidase family U32	941	178	0.189	Actinobacteria, Aquificae, Chlorobi, Deferribacteres, Elusimicrobia, Fibrobacteres, Firmicutes, Fusobacteria, Proteobacteria, Spirochaetes, Tenericutes, Thermotogae
PF00416	Ribosomal protein S13/S18	915	245	0.268	Actinobacteria, Chloroflexi, Deinococcus-Thermus, Dictyoglomi, Firmicutes Gemmatimonadetes, Nitrospirae, Proteobacteria, Spirochaetes, Synergistetes, Tenericutes, Thermobaculum
PF01256, PF03853	Carbohydrate kinase, YjeF-related protein N-terminus	825	210	0.255	Acidobacteria, Actinobacteria, Aquificae, Bacteroidetes, Caldiserica, Chlamydiae, Chloroflexi, Chrysiogenetes, Crenarchaeota, Deferribacteres, Dictyoglomi, Euryarchaeota Firmicutes, Fusobacteria, Ignavibacteriae, Korarchaeota, Nitrospirae, Proteobacteria, Spirochaetes, Thermobaculum., Thermodesulfobacteria, Thermotogae, candidate division NC10
PF01396, PF01131, PF01751	Topoisomerase DNA binding C4 zinc finger, DNA topoisomerase, Toprim domain	823	128	0.156	Acidobacteria, Aquificae, Chlamydiae, Chlorobi, Chloroflexi, Deinococcus-Thermus, Elusimicrobia, Firmicutes, Fusobacteria, Nitrospirae, Planctomycetes, Proteobacteria, Tenericutes, Thermobaculum., Thermotogae, candidate division WWE3
PF13344, PF13242	Haloacid dehalogenase-like hydrolase, HAD-hyrolase-like	808	170	0.210	Actinobacteria, Bacteroidetes, Chloroflexi, Deinococcus-Thermus, Dictyoglomi, Euryarchaeota, Firmicutes, Fusobacteria, Planctomycetes, Proteobacteria, Spirochaetes, Thermobaculum
PF00815	Histidinol dehydrogenase	802	188	0.234	Actinobacteria, Aquificae, Bacteroidetes, Chlorobi, Chrysiogenetes, Cyanobacteria, Deferribacteres, Firmicutes, Fusobacteria, Nitrospirae, Proteobacteria, Spirochaetes, Thermodesulfobacteria, Thermotogae, candidate division NC10
PF00290	Tryptophan synthase alpha chain	781	199	0.255	Acidobacteria, Actinobacteria, Bacteroidetes, Chloroflexi, Cyanobacteria, Elusimicrobia, Euryarchaeota, Firmicutes, Gemmatimonadetes, Nitrospirae, Planctomycetes, Proteobacteria, Spirochaetes, Synergistetes, Thaumarchaeota, Thermobaculum., Thermodesulfobacteria, Thermotogae, Verrucomicrobia, candidate division NC10
PF07991, PF01450	Acetohydroxy acid isomeroreductase, catalytic domain, Acetohydroxy acid isomeroreductase, catalytic domain	779	231	0.297	Acidobacteria, Actinobacteria, Aquificae, Chloroflexi, Chrysiogenetes, Cyanobacteria, Deferribacteres, Deinococcus-Thermus, Dictyoglomi, Firmicutes, Nitrospirae, Planctomycetes, Proteobacteria, Spirochaetes, Thermobaculum, Thermodesulfobacteria, Thermotogae, candidate division NC10
PF00334	Nucleoside diphosphate kinase	772	160	0.207	Chlamydiae, Proteobacteria
PF00478, PF00571	IMP dehydrogenase/GMP reductase domain, CBS domain	762	156	0.205	Acidobacteria, Actinobacteria, Aquificae, Bacteroidetes, Chlorobi, Chloroflexi, Cloacimonetes, Deinococcus-Thermus, Elusimicrobia, Firmicutes, Fusobacteria, Ignavibacteriae, Nitrospirae, Planctomycetes, Proteobacteria, Synergistetes, Tenericutes, Thermobaculum., Thermotogae
PF02696	Uncharacterized ACR, YdiU/UPF0061 family	761	200	0.263	Acidobacteria, Actinobacteria, Bacteroidetes, Chrysiogenetes, Deinococcus-Thermus, Euryarchaeota, Firmicutes, Gemmatimonadetes, Nitrospirae, Planctomycetes, Proteobacteria, Spirochaetes, Verrucomicrobia
PF01817, PF00800	Chorismate mutase type II, Prephenate dehydratase	755	167	0.221	Actinobacteria, Aquificae, Bacteroidetes, Chloroflexi, Chrysiogenetes, Crenarchaeota, Deferribacteres, Deinococcus-Thermus, Dictyoglomi, Elusimicrobia, Firmicutes, Fusobacteria, Nitrospirae, Planctomycetes, Proteobacteria, Spirochaetes, Synergistetes, Thermodesulfobacteria, Verrucomicrobia, candidate division NC10
PF00316	Fructose-1-6-bisphosphatase	750	204	0.272	Acidobacteria, Actinobacteria, Aquificae, Bacteroidetes, Chlorobi, Chloroflexi, Chrysiogenetes, Cyanobacteria, Deferribacteres, Firmicutes, Gemmatimonadetes, Ignavibacteriae, Nitrospirae, Planctomycetes, Proteobacteria, Spirochaetes, Verrucomicrobia, candidate division NC10
PF02421, PF07664, PF07670	Ferrous iron transport protein B, Ferrous iron transport protein B C terminus, Nucleoside recognition	747	166	0.222	Acidobacteria, Actinobacteria, Aquificae, Bacteroidetes, Chlorobi, Cloacimonetes, Cyanobacteria, Deinococcus-Thermus, Euryarchaeota, Firmicutes, Fusobacteria, Gemmatimonadetes, Ignavibacteriae, Planctomycetes, Proteobacteria, Spirochaetes, Thermodesulfobacteria, Verrucomicrobia
PF01070	FMN-dependent dehydrogenase	747	152	0.203	Actinobacteria, Chlamydiae, Chlorobi, Chloroflexi, Crenarchaeota, Cyanobacteria, Deinococcus-Thermus, Euryarchaeota, Firmicutes, Fusobacteria, Ignavibacteriae, Korarchaeota, Proteobacteria, Spirochaetes, Tenericutes, Thaumarchaeota, Thermobaculum.
PF03462, PF00472	PCRF domain, RF-1 domain	733	151	0.206	Acidobacteria, Actinobacteria, Candidatus Saccharibacteria, Candidatus Saccharibacteria., Chlamydiae, Chloroflexi, Chrysiogenetes, Deferribacteres, Dictyoglomi, Elusimicrobia, Firmicutes, Fusobacteria, Gemmatimonadetes, Nitrospirae, Planctomycetes, Proteobacteria, Spirochaetes, Synergistetes, Tenericutes, Thermobaculum., Thermodesulfobacteria, Verrucomicrobia
PF01676, PF06415	Metalloenzyme superfamily, BPG-independent PGAM N-terminus (iPGM_N)	731	134	0.183	Bacteroidetes, Chloroflexi, Euryarchaeota, Firmicutes, Fusobacteria, Proteobacteria, Thermobaculum.
PF00132	Bacterial transferase hexapeptide (six repeats)	728	188	0.258	Acidobacteria, Actinobacteria, Aquificae, Chloroflexi, Chrysiogenetes, Cyanobacteria, Deferribacteres, Dictyoglomi, Euryarchaeota, Firmicutes, Nitrospirae, Proteobacteria, Spirochaetes, Synergistetes, candidate division NC10
PF03144, PF00009	Elongation factor Tu domain 2, Elongation factor Tu GTP binding domain	722	159	0.220	Bacteroidetes, Chlorobi, Chloroflexi, Cyanobacteria, Deferribacteres, Deinococcus-Thermus, Fibrobacteres, Firmicutes, Gemmatimonadetes, Nitrospirae, Planctomycetes, Proteobacteria, Spirochaetes, Verrucomicrobia
PF02233	NAD(P) transhydrogenase beta subunit	720	189	0.263	Actinobacteria, Cyanobacteria, Firmicutes, Proteobacteria

*Multiple protein family ids and descriptions are separated by comma*.

## Discussion

We identified genes that are frequently or non-frequently exchanged among prokaryotic species (Datasets S1, S2). Identification of frequently-transferred proteins among prokaryotes is invaluable for ongoing evolutionary biology and microbiology studies to combat, for example, the widespread antibiotic resistance (Huddleston, 2014) and to better understand bacterial evolution in natural habitats (Smillie et al., 2011). In turn, identification of genes not massively influenced by HGT carries implications for our efforts to reconstruct the deep evolutionary history of cellular organisms (and viruses) by accurate depictions of the tree of life (ToL) using molecular markers that are relatively robust to non-vertical evolution. Our effort thus could become important starting points from both public health and basic science perspectives, as we provide a preliminary list of HGT-related genes in prokaryotes through exhaustive implementations of tree reconstructions and reconciliations in available completely-sequenced prokaryotic genomes.

We refer to the list of HGT genes as “preliminary” since we only studied HGT sensitivity of prokaryotic genes that were distributed in at least 10 but <50% of available genomes. The former criterion was implemented to ensure HGT-indices across some genes were relatively comparable. For example, a gene detected in only four genomes could have two transfers resulting in a higher HGT-index of 0.5. In turn, the latter criterion of excluding very large gene sets comprising of >50% of available genomes in Jeong et al. (2016) could be a major limitation of our study. While, widespread genes are more likely to be inherited vertically (Nasir and Caetano-Anollés, 2013), it is possible that spread of some of these genes was increased laterally over evolutionary timespans leading to their presence in nearly all known prokaryotic species. Exploring the HGT sensitivity of widespread genes, for example, ribosomal proteins, is especially important since it is common practice to concatenate ribosomal proteins in attempts to reconstruct trees of life describing the evolutionary history of cellular organisms (e.g., in Hug et al., 2016). However, dealing with large gene sets followed by large-scale reconstructions and reconciliations is computationally and conceptually challenging (prompting gene concatenation) and that is why, gene sets carrying >50% genomes were excluded during the initial reconstruction of *HGTree* (Jeong et al., 2016). This is however a challenge we are undertaking in an independent study (Jeong et al., unpublished data).

Our approach of HGT detection relies on an explicit evolutionary method of tree reconstruction and reconciliation, generally known to be reliable in HGT detection, especially of ancestral events (Lawrence and Ochman, 2002). The method however differs starkly from some of the composition-based methods, such as evaluating GC and codon biases across genomes to infer HGT (Lawrence and Ochman, 1997, 1998; Ochman et al., 2000), that generally lose effectiveness in detecting HGTs involving longer evolutionary timespans (Lawrence and Ochman, 2002; Ravenhall et al., 2015). Moreover, inferring HGT using different genomic signatures can sometimes lead to different results (Becq et al., 2010). Hence, the composition-based method is perhaps not well suited for large-scale genome-wide global detection of HGT events across thousands of sequenced genomes, and especially, for detection of ancestral gene transfers (Lawrence and Ochman, 2002).

On balance, the phylogenetic method of HGT detection is expected to not perform very well for HGTs occurring between very closely related strains or species that likely do not produce detectable tree incongruence (see Adato et al., 2015 for a possible solution based on the synteny index). In addition, the method, by default, is restricted to genes and protein coding sequences of prokaryotic genomes and precludes detection of transfer of non-coding DNA. Similarly, addition of viral and eukaryotic genomes to the phylogeny-based HGT detection pipeline remains an open challenge since both sets of genomes pose unique challenges. For example, integration of eukaryotic genomes will raise questions regarding the choice of correct “outgroup” to root the reference ToL during tree reconciliation step, a topic that remains controversial (Gouy et al., 2015). Eukaryotic genomes also house large chunks of non-coding sequences that are horizontally acquired such as the acquisition of endogenous viral-like elements (Katzourakis and Gifford, 2010). In turn, viruses are frequent mediators of genetic exchange and there are numerous instances of gene transfer in either direction from cells to viruses and from viruses to cells, albeit the latter seems to be quantitatively dominant as demonstrated by recent large-scale bioinformatics studies (Daubin et al., 2003; Cortez et al., 2009; Nasir and Caetano-Anollés, 2015; Forterre, 2016; Nasir et al., 2017). However, the place of viruses in the ToL is hotly debated (Claverie and Ogata, 2009; Moreira and Lopez-Garcia, 2009), again a concept that is now increasingly been revisited (Nasir et al., 2012; Nasir and Caetano-Anollés, 2015; Claverie and Abergel, 2016; Forterre, 2016). Importantly, viruses do not encode a universal gene marker like 16S or 18S rRNA in cells to be used for the reconstruction of reference species trees. Therefore, virus-mediated gene transfer can perhaps be better studied by utilizing comparative genomics approaches, as in Nasir and Caetano-Anollés (2015), or using protein-folds (Nasir and Caetano-Anollés, 2017) that are relatively more conserved than molecular sequences (Illergård et al., 2009; Abrescia et al., 2012).

Considering the above-mentioned limitations, the number of HGTs detected in our studies are likely to be underestimated. However, no (computational) method of HGT detection is expected to be 100% accurate owing to the inherent complexity underlying the myriad of ways microorganisms interact with each other in natural environments and considering the billions of years of evolutionary timespan for the age of some of the microbial genomes. Therefore, the reported lists of genes that are frequently (Dataset S1) or non-frequently transferred (Dataset S2) among prokaryotic genomes only represent “candidate” HGT genes and should be interpreted that way. We also caution that “orthologous” gene sets vary greatly in species composition and thus HGT-indices across genes may not always be comparable. For example, a gene could be present in 40% available genomes belonging to several microbial phyla and genera indicating high phylogenetic diversity. In comparison, some genes could be narrowly distributed among prokaryotes indicating low phylogenetic diversity. It is logical to think that the number of HGTs would correlate with the phylogenetic diversity of the gene set (as conflicts would become more visible) and thus HGT-index could perhaps further be normalized by the tree length of the reference species tree (or by addition of some other parameters). As counter argument, however, it can be argued that HGTs would increase among organisms that are either closely-related in evolution (expected to harbor a similar mobilome) or reside in close physical proximity (allows greater cell-to-cell contact to facilitate more HGTs) than among microorganisms that are distantly-related either phylogenetically or ecologically (Smillie et al., 2011). In other words, a lot remains to be understood about HGT, especially since the emergence of metagenomic and microbiome datasets that enable a systems biology exploration of microbial life. Considering these complications, we have, for now, restricted the calculation of HGT-index of each gene by simply dividing the total number of detected HGT events on a gene tree by the total number of genomes member of that gene tree. Since, in our opinion, true HGT potential of a gene should be calculated considering both genomes that do and do not participate in its horizontal transfer. In a separate study, we are investigating the phylogenetic vs. ecological effect on the human microbiome dataset using the tree-based HGT-detection method to better understand the “rules” behind increasing/decreasing HGT (Jeong et al., unpublished data). Therefore, our study should be taken as a first step toward meeting the genomics and microbiology needs posed in the post-genomic era. Our lists are preliminary, identify interesting proteins that participate or interfere in antibiotic usage pathways that could have broad utility for evolutionary biologists and microbiologists, and are expected to inspire improved future studies on this topic to better tackle HGT in prokaryotic genomes.

## Materials and methods

Orthologous genes and HGT event mappings were retrieved directly from Jeong et al. (2016). In brief, protein sequences from 2,472 completely-sequenced prokaryotic organisms (156 Archaea and 2,316 Bacteria) were processed using the Ensembl homology prediction pipeline (Vilella et al., 2009) to produce homologous gene sets. Mestortho (ver. 2.0) (Kim et al., 2008) was used to extract orthologous gene sets from homologous clusters. 16S rRNA genes were detected by RNammer (ver. 1.2) (Lagesen et al., 2007). Multiple sequence alignments of orthologous genes and 16S rRNA genes were performed using CLUSTAL-omega (ver. 1.2.1) (Sievers et al., 2014). ML gene and reference trees were reconstructed using FastTree (ver. 2.0) (Price et al., 2010). For 16S rRNA reference trees, 18S rRNA sequence from *Saccharomyces cerevisiae* was added and treated as “outgroup” to root the reference tree. Gene and reference trees were reconciled using RANGER-DTL-U algorithm (ver. 1.0) (Bansal et al., 2012). Protein families (Finn et al., 2014) were assigned to orthologous gene sets based on the 80% “majority” rule [i.e., when 80% of proteins in a given gene set yielded the same Pfam ID when scanned via the PfamScan (ver. 1.3) algorithm]. Molecular functions, biological processes, and cellular component descriptions, as defined by the GO database (Ashburner et al., 2000). HGT events on gene trees were compared using in-house Python scripts. The impact of multiple equally parsimonious reconciliations between gene and reference trees and their influence on resulting duplication, transfer, or loss event assignments was not explored. Similarly, the choice of root among equally parsimonious rootings of gene tree when reconciling against rooted species tree was also not evaluated. Both due to computational infeasibility considering the size of our dataset and available computational resources.

## Author contributions

Both authors contributed to the design and analysis of the study and wrote, edited, and approved the final manuscript.

### Conflict of interest statement

The authors declare that the research was conducted in the absence of any commercial or financial relationships that could be construed as a potential conflict of interest.

## Supplementary material

### Deposited data and information to the user

Datasets S1, S2 have been deposited to figshare and can be retrieved at https://figshare.com/s/ab90c3e792d651a61d39. Users can download and reuse the data for research purpose only with an acknowledgment to us and citing this paper and the original *HGTree* study (Jeong et al., 2016).

Dataset S1 A list of 69,365 candidate HGT-genes. Each gene set is given a numeric Id. The second column gives accession numbers for all proteins member of the gene set. Protein family assignments and GO annotations for biological processes, cellular component, and molecular function GO terms are listed next to each gene set. Multiple Pfam and GO hits are separated by comma. Pfam assignments rely on at least 80% of genes in any orthologous gene set matching the same Pfam Id. HGT-index is the fraction of total detected HGT events in each gene tree divided by the total number of taxa (genomes) in that gene tree. Data sorted by HGT-index in a descending manner.

Dataset S2 A list of 47,980 candidate HGT-free genes. Each gene set is given a numeric Id. The second column gives accession numbers for all proteins member of the gene set. Protein family assignments and GO annotations for biological processes, cellular component, and molecular function GO terms are listed next to each gene set. Multiple Pfam and GO hits are separated by comma. Pfam assignments rely on at least 80% of genes in any orthologous gene set matching the same Pfam Id.
